# Efficacy and Safety of Bone Marrow-Derived Mesenchymal Stem Cells for Chronic Antibody-Mediated Rejection After Kidney Transplantation- A Single-Arm, Two-Dosing-Regimen, Phase I/II Study

**DOI:** 10.3389/fimmu.2021.662441

**Published:** 2021-06-25

**Authors:** Yongcheng Wei, Xiaoyong Chen, Huanxi Zhang, Qun Su, Yanwen Peng, Qian Fu, Jun Li, Yifang Gao, Xirui Li, Shicong Yang, Qianyu Ye, Huiting Huang, Ronghai Deng, Gang Li, Bowen Xu, Chenglin Wu, Jiali Wang, Xiaoran Zhang, Xiaojun Su, Longshan Liu, Andy Peng Xiang, Changxi Wang

**Affiliations:** ^1^ Organ Transplant Center, the First Affiliated Hospital, Sun Yat-sen University, Guangzhou, China; ^2^ Center for Stem Cell Biology and Tissue Engineering, Key Laboratory for Stem Cells and Tissue Engineering, Ministry of Education, Sun Yat-Sen University, Guangzhou, China; ^3^ The Biotherapy Center, The Third Affiliated Hospital, Sun Yat-sen University, Guangzhou, China; ^4^ Department of Pathology, The First Affiliated Hospital, Sun Yat-sen University, Guangzhou, China; ^5^ Department of Nephrology, The First Affiliated Hospital, Sun Yat-sen University, Guangzhou, China

**Keywords:** mesenchymal stem cells, kidney transplantation, antibody-mediated allograft rejection, stem cell therapy, alloimmunity

## Abstract

**Objective:**

To investigate the efficacy and safety of bone marrow-derived mesenchymal stem cells (BM-MSCs) on chronic active antibody-mediated rejection (cABMR) in the kidney allograft.

**Methods:**

Kidney recipients with biopsy-proven cABMR were treated with allogeneic third-party BM-MSCs in this open-label, single-arm, single-center, two-dosing-regimen phase I/II clinical trial. In Regimen 1 (n=8), BM-MSCs were administered intravenously at a dose of 1.0×10^6^ cells/kg monthly for four consecutive months, while in Regimen 2 (n=15), the BM-MSCs dose was 1.0×10^6^ cells/kg weekly during four consecutive weeks. The primary endpoints were the absolute change of estimated glomerular filtration rate (eGFR) from baseline (delta eGFR) and the incidence of adverse events associated with BM-MSCs administration 24 months after the treatment. Contemporaneous cABMR patients who did not receive BM-MSCs were retrospectively analyzed as the control group (n =30).

**Results:**

Twenty-three recipients with cABMR received BM-MSCs. The median delta eGFR of the total BM-MSCs treated patients was -4.3 ml/min per 1.73m^2^ (interquartile range, IQR -11.2 to 1.2) 2 years after BM-MSCs treatment (P=0.0233). The median delta maximum donor-specific antibody (maxDSA) was -4310 (IQR -9187 to 1129) at 2 years (P=0.0040). The median delta eGFR of the control group was -12.7 ml/min per 1.73 m^2^ (IQR -22.2 to -3.5) 2 years after the diagnosis, which was greater than that of the BM-MSCs treated group (P=0.0342). The incidence of hepatic enzyme elevation, BK polyomaviruses (BKV) infection, cytomegalovirus (CMV) infection was 17.4%, 17.4%, 8.7%, respectively. There was no fever, anaphylaxis, phlebitis or venous thrombosis, cardiovascular complications, or malignancy after BM-MSCs administration. Flow cytometry analysis showed a significant decreasing trend of CD27^-^IgD^-^ double negative B cells subsets and trend towards the increase of CD3^+^CD4^+^PD-1^+^/lymphocyte population after MSCs therapy. Multiplex analysis found TNF-α, CXCL10, CCL4, CCL11 and RANTES decreased after MSCs treatment.

**Conclusion:**

Kidney allograft recipients with cABMR are tolerable to BM-MSCs. Immunosuppressive drugs combined with intravenous BM-MSCs can delay the deterioration of allograft function, probably by decreasing DSA level and reducing DSA-induced injury. The underlying mechanism may involve immunomodulatory effect of MSCs on peripheral B and T cells subsets.

## Introduction

Kidney transplantation is the most effective treatment for end-stage renal disease (ESRD) as it can significantly prolong life expectancy and improve life quality of patients with ESRD. Over the last decades, the 1-year graft survival rate has increased to nearly 95%. However, long-term survival is still unsatisfactory, and the 10-year graft survival rate is nearly 60% (1). Antibody-mediated rejection (ABMR) is the main cause of long-term graft loss after kidney transplantation (2). B cells can be activated under many circumstances prior to or after transplantation to produce donor-specific antibodies (DSA) which are mainly anti-HLA (human leukocyte antigen) antibodies. The DSA attack vascular endothelium of renal allografts through complement-dependent cytotoxicity (CDC) and antibody-dependent cellular cytotoxicity (ADCC) pathways, causing microvascular inflammation and leading, to chronic damage, in the long-term including transplant glomerulopathy, arterial intimal thickening, renal tubular atrophy and interstitial fibrosis, etc (3).

ABMR, which presents largely heterogenic clinical manifestation, can be pathologically classified into active ABMR and chronic active ABMR (cABMR), according to 2019 Banff classification criteria (4). Patients with cABMR often present a gradual increment in serum creatinine with or without deteriorative proteinuria months or even years after the development of *de novo* DSA. Contrary to the improvement achieved on diagnostic techniques and mechanism understanding, there is still a lack of efficient treatment for cABMR. In this sense, a comprehensive treatment strategy involving the administration of plasmapheresis (PP) and/or intravenous immunoglobulin (IVIG), combing with rituximab, bortezomib or daclizumab, etc. is currently being used. Unfortunately, most studies on these treatments are small, lack solid evidence, and the reported findings are often inconsistent or even opposite (5). Many patients with cABMR ultimately progress to renal allograft failure despite receiving a strong comprehensive treatment, which increases medical cost and risk of infection. Therefore, there is an urgent need to develop novel therapeutic strategies for cABMR treatment.

Mesenchymal stem cells (MSCs) are widely distributed in various tissues and organs of the human body and exert immunomodulatory potential with low immunogenicity (6). Since 2004, when MSCs were introduced to treat graft versus host disease (GvHD) after bone marrow transplantation (7), its therapeutic effect on inflammatory and autoimmune diseases have been widely reported (8). Multiple studies have demonstrated the potential effect of MSCs in kidney transplantation as induction therapy, minimizing calcineurin inhibitors (CNIs), rejection treatment and tolerance induction (9–15). In addition, preclinical studies have showed that MSCs therapy prevents interstitial fibrosis and tubular atrophy in a chronic rejection model of rat kidney transplantation (16, 17). Moreover, a phase I study indicates that MSCs are clinically feasible and safe treatment for subclinical rejection and interstitial fibrosis in kidney transplantation (18). These findings suggest that MSCs may have protective effects on renal allograft function in the setting of chronic rejection. Despite its overall safety, reported in many studies, MSCs infusion has also been reported to increase serum creatinine, drawing the attention to its adverse effects in kidney transplantation (19). Nevertheless, the efficacy and safety of MSCs to treat cABMR in kidney transplantation has not been well investigated. In this prospective two-dosing-regimen phase I/II clinical trial, MSCs were intravenously administered to 23 biopsy-proven cABMR patients.The renal allograft function, DSA level and adverse events were subsequently assessed during 24 months after treatment. The mechanism involved in the effect of MSCs in patients with cABMR was also explored.

## Patients and Methods

### Study Design and Participants

We designed an open-label, single-arm, single-center, two-dosing-regimen, phase I/II clinical trial with a 24-month follow-up period. The study was conducted in the First Affiliated Hospital of Sun Yat-sen University between 2013 and 2020. Kidney recipients with histological-proven cABMR, with presence or absence of circulating donor-specific anti-human leukocyte antigen (HLA) antibodies (DSA), between 18 and 65 years old were eligible for the study. Histological evaluation of renal biopsies was performed in accordance with Banff criteria (4). The patients were administered with a maintenance immunosuppressive regimen consisting of calcineurin inhibitors, mycophenolate with or without glucocorticoids, and were treated with BM-MSCs as the initial regimen or second-line regimen after previous failed ABMR treatment, including plasmapheresis, intravenous immunoglobulin, rituximab, bortezomib and methylprednisolone (**Supplemental Table 1**). The exclusion criteria included pregnancy, combined organ transplantation, active infection, white blood cell count < 3×10^9^/L, hemoglobin < 50 g/L, and severe cardiovascular or gastrointestinal complications. This study was approved by the Ethics Committee of the First Affiliated Hospital of Sun Yat-sen University and was conducted in accordance with the principles of the World Medical Association Declaration of Helsinki. Written informed consent was obtained from all the participants.

For comparison with the BM-MSCs treated group, adult kidney recipients (≥18 years old) who were histologically diagnosed as cABMR in the same transplant center between 2013 and 2020 were retrospectively enrolled as the contemporaneous control group. Patients with a baseline eGFR lower than 15 ml/min per 1.73m^2^ at the time of diagnosis were excluded.

### Procedures

Between 2013 and 2015, BM-MSCs were administered intravenously at a dose of 1.0×10^6^ cells/kg per month for four consecutive months (Regimen 1). Since 2016, a strengthened therapy was used. BM-MSCs were intravenously infused at a dose of 1.0×10^6^ cells/kg per week for four consecutive weeks (Regimen 2). The BM-MSCs were added to 30 ml sterilized normal saline and infused within 30 minutes. The primary endpoints were the absolute change of estimated glomerular filtration rate (eGFR) from baseline, and the incidence of adverse events (AEs) associated with BM-MSCs administration 24 months after BM-MSCs treatment. eGFR was calculated by the modification of diet in renal disease (MDRD) equation. The secondary endpoints included absolute change of maximum DSA (maxDSA) mean fluorescence intensity (MFI) level from baseline, and patient and graft survival. maxDSA is defined as the DSA loci with the highest MFI value. In Regimen 1, the patients were evaluated at screening, monthly during the first 4 months, and then at 6, 12 and 24 months after BM-MSCs treatment. In Regimen 2, the patients were examined at screening, weekly during the first month, and then at 1, 3, 6, 12 and 24 months after BM-MSCs treatment. Routine laboratory tests and adverse events monitoring were performed at every visit. DSA MFI level was measured at 1, 6, 12 and 24 months. Blood samples were collected at every visit and stored for further examinations. In the control group, the demographic, clinical, laboratory, treatment and outcome data in the following two years after diagnosis were collected from medical records.

### Isolation and Characterization of BM-MSCs

Human bone marrow samples donated from four male and two female third-party healthy donors were used to isolate and expand BM-MSCs, after informed consent. All the donors were negative for human immunodeficiency virus (HIV), hepatitis B virus (HBV), hepatitis C virus (HCV), human T cell virus, human herpes virus (HTLV), Epstein-Barr virus (EBV), human cytomegalovirus (HCMV), and treponema pallidum (TP). The age of the healthy donors was 27.8 ± 5.8 years old. The donors were healthy and did not presented hematopoietic, genetic, autoimmune diseases, nor mental disorders.

The bone marrow was diluted 1:1 with human MSCs culture medium (Xeno-free media, GIBCO, Grand Island, NY, USA). Bone marrow mononuclear cells were separated by Ficoll-Paque (1.077 g/mL; GE Healthcare Life Sciences, Little Chalfont, Buckinghamshire, UK) using density gradient centrifugation and were seeded at a density of 1x10^5^ cells/cm^2^ in T75 cell culture flasks. When 80% confluence was reached, the cells were detached using Trypsin-EDTA (0.25%, GIBCO, Grand Island, NY, USA) and were designated as passage 1. These cells were further passaged at a ratio of 1:3. The culture-expanded BM-MSCs exhibit fibroblast-like (spindle and fusiform) and uniform morphology in adherent cultures, expressed CD29, CD44, CD73, CD90, CD105 and CD166, but not CD34 or CD45 (**Supplemental Figure 1**). They were able to differentiate into osteoblasts, adipocytes and chondroblasts under standard *in vitro* differentiating conditions.

BM-MSCs at passage 7 to 8 were used for the treatments described in the study. The selection criteria of the BM-MSCs used in the trial includes: presence of normal karyotypes; cell viability greater than 95%; absence of visible clumps; sterility: negative for fungi, bacteria, mycoplasma, HIV, HBV, HCV, HTLV, EBV, HCMV, TP and endotoxin; and cell purity: their expression of CD29, CD44, CD73, CD90, CD105 and CD166 must be above 95% and must lack expression of CD34 and CD45 (<2%).

### Peripheral Blood Lymphocyte Immunophenotyping

Principal lymphocyte subsets of monocytes, B lymphocytes, T lymphocytes, dendritic cells, regulatory T cells and TCR αβ and TCR γδ protein chain expression, and detection of Vδ1+ and Vδ2+ subsets among TCRγδ+ T cells, were assessed by flow-cytometry using the following DuraClone panels (Beckman Coulter, Miami, FL, USA): IM Phenotyping Basic panel, IM B cell panel, IM T Cell Subsets panel, IM Dendritic Cell panel, IM Treg panel and IM TCRs panel (**Supplemental Table 2**).

Sample preparation was performed according to the manufacturer’s instructions. Sample acquisition was conducted on an eight-color Navios flow cytometer and the data generated were analyzed using Kaluza Analysis 2.1 software (Beckman Coulter, Miami, FL, USA). Representative figures depicting the gating strategy are shown in **Supplemental Figure 2**.

### Detection of Anti-HLA Antibody

Anti-HLA antibody profiling of serum from kidney recipients was performed according to the instruction of LIFECODES^®^ LSA™ Class I/II kit (Immucor GTI Diagnostics, Inc. Waukesha, WI, USA). Briefly, Millipore multiscreen filter plate wells were pre-wet with distilled water for five minutes, then the water was removed. The LSA™ Beads were briefly centrifugated and then thoroughly vortexed for one minute. Then, 40 µl of LSA™ Beads were added to each of the assigned wells. Re-vortex intermittently and add 5 µl of patient serum and control sera and mix well. Then were incubate for 30 minutes on a rotating platform in the dark and at room temperature. After incubation, the beads were resuspended and washed three times with Wash Buffer. Then, 50 µl of 1 X conjugate was added to each well, placed on a rotating platform for 10 minutes, and incubate for 30 minutes in dark. Finally, 150 µl of Wash Buffer were added to resuspend the beads, and the solution was read on the Luminex^®^ 200 platform. The result were analyzed with the LIFECODES^®^ MatchIT! Antibody software (Immucor GTI Diagnostics, Inc. Waukesha, WI, USA). Mean fluorescence intensity (MFI)>1000 was considered positive.

### Detection of Serum Cytokines and Chemokines

Serum cytokines and chemokines were assessed according to the instruction of Luminex^®^ Assay Human Premixed Multi-Analyte Kit (R&D Systems, Inc. Minneapolis, MN, USA). The kit detects BAFF, CCL3, CCL11, CXCL10, IFN-γ, IL-2, IL-5, IL-8, IL-12 p70, CCL2, CCL4, CXCL1, GM-CSF, IL-1β, IL-4, IL-6, IL-10, IL-13, TNF-α, CXCL12 and RANTES. Briefly, 50 µl of standard or sample was added to each well of a microplate followed by the addition of 50 µl of diluted Microparticle Cocktail. The microplate was then covered with a foil plate sealer and incubated on a horizontal orbital microplate shaker at 800 rpm at room temperature for two hours. The plate was washed three times with Wash Buffer. Then, 50 µl of diluted Biotin-Antibody Cocktail was added to each well and incubated again on a horizontal orbital microplate shaker at 800 rpm at room temperature for one hour with a foil plate sealer. After that, the plate was washed 3 times, 50 µl of diluted Streptavidin-PE were added to each well, and the plate was incubated on a horizontal orbital microplate shaker at 800 rpm at room temperature for 30 minutes with a foil plate sealer. Finally, the plate was washed three times, 100 µl of Wash Buffer were added to each well, and the plate was incubated for 2 minutes at room temperature. The samples were then read in a Luminex^®^ MAGPIX^®^ analyzer (R&D Systems, Inc. Minneapolis, MN, USA).

### Statistical Analysis

Continuous data with normal distribution was presented as mean ± standard deviation (SD) and compared using Student’s t-test, while continuous data without normal distribution was expressed as median (lower quartile-higher quartile) and compared using Mann-Whitney U test. Categorical data was reported as counts and percentages and compared using Chi-square test or Fisher’s exact test. Kaplan-Meier method was used for survival analysis and the log-rank test was used to compare two survival curves. Paired t-test or Wilcoxon signed-rank test was used to analyze paired differences. The linear trend test was used to determine the significance of trends over time. Multivariate linear regression was performed to adjust for confounding factors affecting the decline rate of eGFR. Last observation carried forward (LOCF) was used for data imputation (20). Significance was set at *p* < 0.05. Statistical analyses were performed using GraphPad Prism 8.0.1 software (GraphPad Corporation, La Jolla, CA, USA) and ‘R’, a free software environment for statistical computing and graphics (www.r-project.org).

## Results

### Baseline Characteristics of Kidney Transplant Recipients With Chronic Antibody-Mediated Rejection

The baseline characteristics of the 23 kidney recipients with BM-MSCs treatment were shown in **Table 1**. Eight patients were enrolled in Regimen 1 and fifteen in Regimen 2. The baseline characteristics between patients enrolled in Regimen 1 and Regimen 2 were not significantly different. Regarding combined histological lesions, there were two cases of IgA nephropathy (IgAN) (8.7%), one of CNI nephrotoxicity (4.3%), and one of borderline rejection (4.3%); all the cases were in Regimen 2 group. In addition, no acute/chronic T cell-mediated rejection or acute ABMR were identified. Other medications used for cABMR before or after MSCs treatment are listed in **Supplemental Table 1**. The histological scores at the time of cABMR diagnosis, summarized in **Table 2**, were similar between Regimen 1 and Regimen 2.

**Table 1:** Baseline characteristics of chronic antibody-mediated rejection patients before BM-MSCs treatment and the contemporaneous control.

Characteristics	Total	MSCs			Control	P value^*^	P value^†^
		Total	Regimen 1	Regimen 2			
Patient number (n)	53	23	8	15	30		
Age (years), mean (SD)	45.5 (11.3)	42.9 (11.0)	40.6 (12.9)	44.07 (10.1)	47.4 (11.4)	0.4869	0.1473
Gender						0.3452	0.2352
Male, No. (%)	41 (77.4%)	16 (69.6%)	7 (87.5%)	9 (60.0%)	25 (83.3%)		
Female, No. (%)	12 (22.6%)	7 (30.4%)	1 (12.5%)	6 (40.0%)	5 (16.7%)		
BMI (kg/m^2^), mean (SD)	22.6 (3.9)	22.3 (3.6)	21.2 (3.7)	22.9 (3.6)	22.9 (4.1)	0.2943	0.6113
Baseline eGFR (ml/min/1.73m^2^), mean (SD)	38.3 (18.3)	42.6 (19.0)	48.0 (22.6)	39.7 (16.8)	35.1 (17.4)	0.3296	0.1412
Baseline maxDSA MFI^※^, mean (SD)	12103 (5750)	12554 (4164)	11357 (4215)	13153 (4189)	11750 (6813)	0.4048	0.6624
Time from transplantation to treatment (years), mean (SD)		8.5 (5.7)	4.8 (2.8)	10.5 (6.0)		0.0191	
Maintenance immunosuppressants, No. (%)						0.5576	0.1472
Tacrolimus + MPA + Steroids	40 (75.5%)	21 (91.3%)	8 (100%)	13 (86.7%)	19 (63.3%)		
Tacrolimus + MPA + Steroids + Rapamycin	2 (3.8%)	0 (0)			2 (6.7%)		
Cyclosporine A + MPA + Steroids	8 (15.1%)	1 (4.3%)	0 (0)	1 (6.7%)	7 (23.3%)		
Tacrolimus + MPA (Steroids free)	2 (3.8%)	1 (4.3%)	0 (0)	1 (6.7%)	1 (3.3%)		
Cyclosporine A + MPA (Steroids free)	1 (1.9%)	0 (0)			1 (3.3%)		
Combined histological lesions						0.4921	0.6729
IgAN, No. (%)	5 (9.4%)	2 (8.7%)	0 (0)	2 (13.3%)	3 (10%)		
CNI nephrotoxicity, No. (%)	6 (11.3%)	1 (4.3%)	0 (0)	1 (6%)	5 (16.7%)		
Borderline rejection, No. (%)	3 (5.7%)	1 (4.3%)	0 (0)	1 (6%)	2 (6.7%)		
Other, No. (%)	2 (3.8%)	1 (4.3%)	0 (0)	1 (6%)	1 (3.3%)		

BM-MSCs, bone marrow derived mesenchymal stem cells; BMI, body mass index; Baseline maxDSA MFI^※^, maximum donor-specific antibody mean fluorescence intensity pre-MSCs treatment or at diagnosis; Scr, serum creatinine; eGFR, estimated glomerular filtration rate; SD, standard deviation; IgAN, IgA nephropathy; CNI, calcineurin inhibitors; MPA, mycophenolate acid. ^*^P value between Regimen 1 and Regimen 2. ^†^P value between total BM-MSCs treatment group and contemporaneous control.

**Table 2:** Baseline Banff scores at the time of BM-MSCs treatment initiation for the BM-MSCs group, or at the time of histologic diagnosis of cABMR for the contemporaneous control.

Total (N = 53), No. (%)		MSCs			Control (N = 30), No. (%)	P value^*^	P value^†^
		Total (N = 23), No. (%)	Regimen 1 (N = 8), No. (%)	Regimen 2 (N = 15), No. (%)		
Glomerulitis (g)						0.9802	0.6246
1	15 (28.3%)	6 (26.1%)	2 (25.0%)	4 (26.7%)	9 (30.0%)		
2	21 (39.6%)	8 (34.8%)	3 (37.5%)	5 (33.3%)	13 (43.3%)		
3	17 (32.1%)	9 (39.1%)	3 (37.5%)	6 (40.0%)	8 (26.7%)		
Peritubular capillaritis (ptc)						0.2136	0.3614
0	3 (5.7%)	2 (8.7%)	0 (0)	2 (13.3%)	1 (3.3%)		
1	18 (34.0%)	5 (21.7%)	2 (25.0%)	3 (20.0%)	13 (43.3%)		
2	25 (47.2%)	12 (52.2%)	6 (75.0%)	6 (40.0%)	13 (43.3%)		
3	7 (13.2%)	4 (17.4%)	0 (0)	4 (26.7%)	3 (10.0%)		
Tubulitis (t)						0.6077	0.4726
0	23 (43.4%)	9 (39.1%)	4 (50.0%)	5 (33.3%)	14 (46.7%)		
1	29 (54.7%)	13 (56.5%)	4 (50.0%)	9 (60.0%)	16 (53.3%)		
3	1 (1.9%)	1 (4.3%)	0 (0)	1 (6.7%)	0 (0)		
Intimal or transmural arteritis (v)						0.2137	0.4312
0	48 (90.6%)	20 (87.0%)	6 (75.0%)	14 (93.3%)	28 (93.3%)		
1	5 (9.4%)	3 (13.0%)	2 (25.0%)	1 (6.7%)	2 (6.7%)		
Inflammation (i)						0.5396	0.3362
0	41 (77.4%)	16 (69.6%)	5 (62.5%)	11 (73.3%)	25 (83.3%)		
1	11 (20.8%)	6 (26.1%)	3 (37.5%)	3 (20.0%)	5 (16.7%)		
3	1 (1.9%)	1 (4.3%)	0 (0)	1 (6.7%)	0 (0)		
Double contour (cg)						0.8485	0.9503
1	15 (28.3%)	7 (30.4%)	3 (37.5%)	4 (26.7%)	8 (26.7%)		
2	17 (32.1%)	7 (30.4%)	2 (25.0%)	5 (33.3%)	10 (33.3%)		
3	21 (39.6%)	9 (39.1%)	3 (37.5%)	6 (40.0%)	12 (40.0%)		
Interstitial fibrosis (ci)						0.1501	0.6907
0	3 (5.7%)	2 (8.7%)	0 (0)	2 (13.3%)	1 (3.3%)		
1	32 (60.4%)	14 (60.9%)	7 (87.5%)	7 (46.7%)	18 (60.0%)		
2	17 (32.1%)	7 (30.4%)	1 (12.5%)	6 (40.0%)	10 (33.3%)		
3	1 (1.9%)	0 (0)	0 (0)	0 (0)	1 (3.3%)		
Tubular atrophy (ct)						0.1501	0.2901
0	2 (3.8%)	2 (8.7%)	0 (0)	2 (13.3%)	0 (0)		
1	31 (58.5%)	14 (60.9%)	7 (87.5%)	7 (46.7%)	17 (56.7%)		
2	19 (35.8%)	7 (30.4%)	1 (12.5%)	6 (40.0%)	12 (40.0%)		
3	1 (1.9%)	0 (0)	0 (0)	0 (0)	1 (3.3%)		
Fibrous intimal thickening (cv)						0.1468	0.0556
0	8 (15.1%)	6 (26.1%)	0 (0)	6 (40.0%)	2 (6.7%)		
1	24 (45.3%)	12 (52.2%)	6 (75.0%)	6 (40.0%)	12 (40.0%)		
2	19 (35.8%)	4 (17.4%)	2 (25.0%)	2 (13.3%)	15 (50.0%)		
3	2 (3.8%)	1 (4.3%)	0 (0)	1 (6.7%)	1 (3.3%)		
C4d staining						0.2516	0.9979
0	21 (39.6%)	9 (39.1%)	1 (12.5%)	8 (53.3%)	12 (40.0%)		
1	9 (17.0%)	4 (17.4%)	2 (25.0%)	2 (13.3%)	5 (16.7%)		
2	11 (20.8%)	5 (21.7%)	3 (37.5%)	2 (13.3%)	6 (20.0%)		
3	12 (22.6%)	5 (21.7%)	2 (25.0%)	3 (20.0%)	7 (23.3%)		

^*^P value between Regimen 1 and Regimen 2. ^†^P value between total BM-MSCs treatment group and contemporaneous control.

### Renal Allograft Function

**Figure 1:**
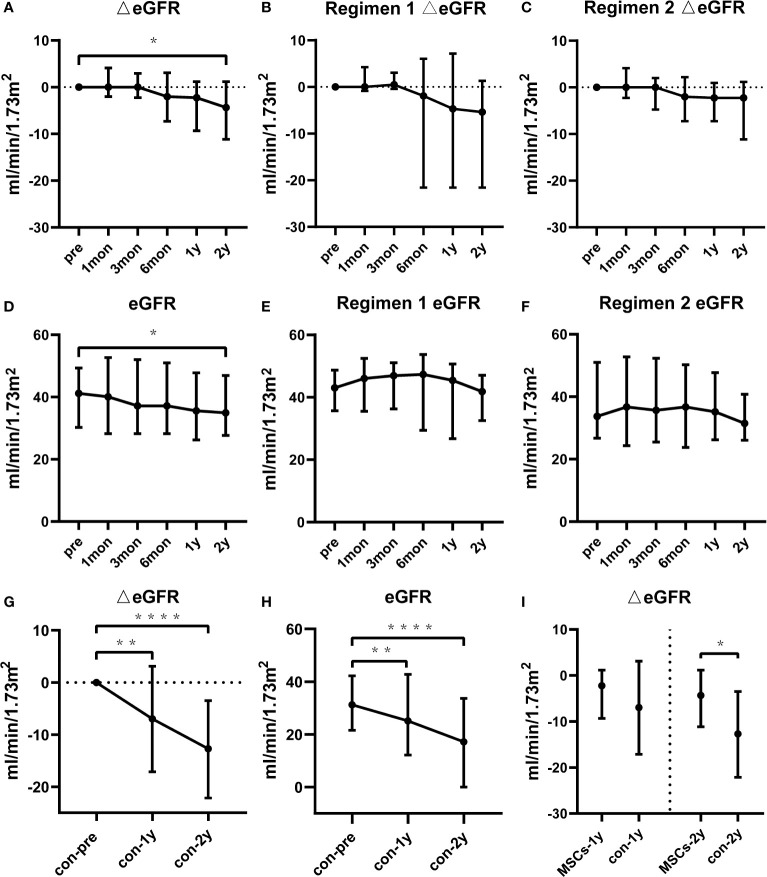
Renal function of the BM-MSCs treated group and the contemporaneous control group in two years of follow-up. Delta estimated glomerular filtration rate (eGFR) pre and after BM-MSCs treatment in total BM-MSCs treated **(A)**, Regimen 1 **(B)**, and Regimen 2 populations **(C)**. eGFR pre and after BM-MSCs treatment in total BM-MSCs treated **(D)**, Regimen 1 **(E)**, and Regimen 2 populations **(F)** during the study period. Delta eGFR of the control group two years after histological diagnosis **(G)**. eGFR of the control group two years after histological diagnosis **(H)**. Comparison of delta eGFR between BM-MSCs treated group and the control group **(I)**. Points represent median. Bars represent the lower quartile and upper quartile. Data before and after BM-MSCs treatment or histological diagnosis were analyzed by Wilcoxon test. Data comparing BM-MSCs-treated and control groups were analyzed by Mann-Whitney test. *p < 0.05, **p < 0.01, ****p < 0.0001.

**Figures 1A–C** shows the changes in estimated glomerular filtration rate (eGFR) for the total study population, Regimen 1 and Regimen 2 patients, respectively. Delta eGFR did not significantly change in one year. At one and two years after BM-MSCs treatment, the median delta eGFR of the total participants was -2.2 ml/min per 1.73m^2^ (IQR -9.3 to 1.2, P>0.05) and -4.3 ml/min per 1.73m^2^ (IQR -11.2 to 1.2, P=0.0233), respectively. Moreover, one year after BM-MSCs treatment, the median delta eGFR was -4.7 ml/min per 1.73m^2^ (IQR -21.6 to 7.1, P=0.3828) in Regimen 1 group and -2.2 ml/min per 1.73m^2^ (IQR -7.3 to 0.9, P=0.0554) in Regimen 2 group. At two years after treatment, the median delta eGFR was -5.4 ml/min per 1.73m^2^ (IQR -21.6 to 1.3, P=0.1953) in Regimen 1 group and -2.2 ml/min per 1.73m^2^ (IQR -11.2 to 1.2, P=0.0833) in Regimen 2 group. eGFR remained stable in one year (**Figures 1D–F**). The median eGFR of the total participants, and those from Regimen 1 and Regimen 2 one year after MSCs treatment was 35.6 ml/min per 1.73m^2^ (IQR 26.3 to 47.8), 45.4 ml/min per 1.73m^2^ (IQR 26.7 to 50.7) and 35.2 ml/min per 1.73m^2^ (IQR 26.3 to 47.7), respectively. This parameter was respectively 34.9 ml/min per 1.73m^2^ (IQR 27.7 to 46.9), 41.8 ml/min per 1.73m^2^ (IQR 32.5 to 47.1) and 31.5 ml/min per 1.73m^2^ (IQR 26.1 to 40.8) two years after BM-MSCs treatment.

### DSA Changes

**Figure 2:**
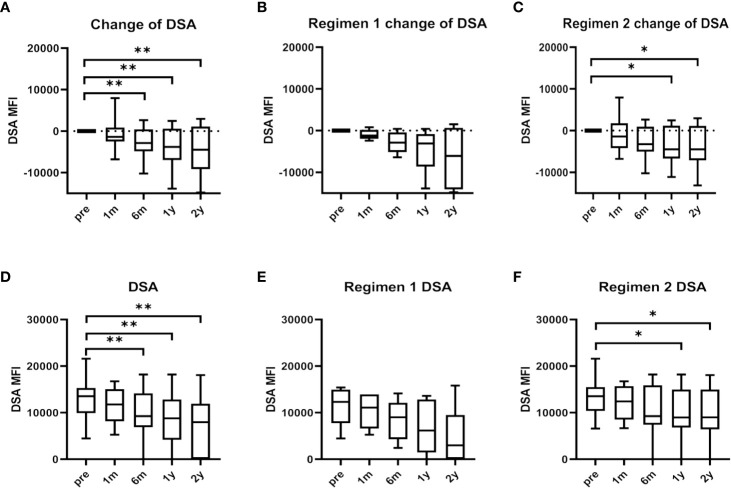
Maximum donor-specific antibody (maxDSA) value pre and after BM-MSCs treatment. Change of maxDSA MFI pre and after BM-MSCs treatment in total BM-MSCs treated **(A)**, Regimen 1 **(B)**, and Regimen 2 populations **(C)**. MaxDSA MFI pre and after BM-MSCs treatment in total BM-MSCs treated **(D)**, Regimen 1 population **(E)**, and Regimen 2 population **(F)** during the study period. Boxplots represent the lower quartile, median and upper quartile. Bars represent minimum and maximum. Data were analyzed by Wilcoxon test. *p < 0.05, **p < 0.01.

Serum maxDSA intensity of the total BM-MSCs population tended to decline six months after BM-MSCs infusions (**Figures 2A, D**). Median serum maxDSA decreased from 13534 to 8797 one year after treatment, and to 7952 two years after MSCs treatment. The median delta maxDSA was -4310 (IQR -9187 to 1129) in the second year (P=0.0040). In patients from Regimen 1, serum maxDSA tended to decline (**Figures 2B, E**), while in Regimen 2 patients maxDSA significantly decreased after one and two years of the first dose of BM-MSCs (**Figures 2C, F**).

### Patient and Graft Survival

**Figure 3:**
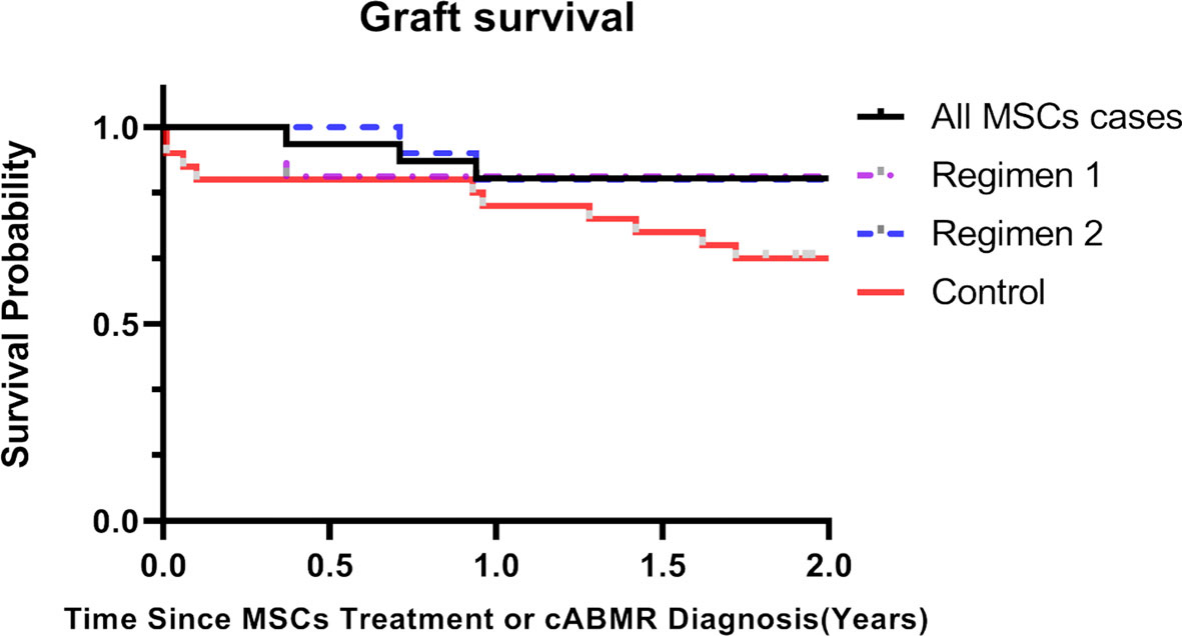
Kaplan-Meier curves of kidney graft survival for chronic antibody-mediated rejection (cABMR) patients after BM-MSCs infusion or histological diagnosis within two years. Kaplan-Meier of graft survival of the total BM-MSCs treated (black), Regimen 1 (purple), and Regimen 2 populations (blue), and of the control group after histological diagnosis of cABMR (red).

No patient died in two years after BM-MSCs treatment. Graft loss was observed in three patients after BM-MSCs infusion within two years of follow-up. One graft loss was detected in Regimen 1 group and two in Regimen 2. The graft survival rate after two years of BM-MSCs treatment was 87.0% (**Figure 3**).

### Adverse Events

We assessed the adverse events (AEs) associated with BM-MSCs administration. Two years after BM-MSCs infusion, one patient presented severe bacterial pneumonia and recovered after receiving appropriate medication, which was considered to be unrelated to the BM-MSCs treatment (**Table 3**). Elevated hepatic enzyme was observed in four patients, three of which resulted from replication of pre-existing hepatitis B virus (HBV) or hepatitis C virus (HCV). Several BK polyomaviruses infections and cytomegalovirus infections were observed and were solved with treatment. Furthermore, no fever, anaphylaxis, phlebitis or venous thrombosis, cardiovascular complications or malignancy were identified during this study.

**Table 3:** Adverse events of cABMR patients treated with BM-MSCs.

Adverse event, No. (%)	Total, (n = 23)	Regimen 1, (n = 8)	Regimen 2, (n = 15)
Hepatic enzyme elevation	4 (17.4%)	3 (37.5%)	1 (6.7%)
Bacterial pneumonia	1 (4.3%)	0 (0)	1 (6.7%)
Polyoma BK virus infection	4 (17.4%)	2 (25.0%)	2 (13.3%)
Cytomegalovirus infection	2 (8.7%)	0 (0)	2 (13.3%)

### Effect of Combined Medications

In order to exclude confounding by other combined medications for ABMR, subgroup analysis was conducted. All BM-MSCs treated patients were divided into BM-MSCs alone subgroup and BM-MSCs combined with other treatments subgroup. Baseline eGFR and maxDSA were similar in these two subgroups. Two years after BM-MSCs treatment, the median delta eGFR of BM-MSCs alone subgroup was similar with that of the BM-MSCs combined with other treatments subgroup [median, -3.8 ml/min per 1.73m^2^ (IQR -10.7 to 1.4) *vs* -4.3 ml/min per 1.73m^2^ (IQR -20.9 to 0.9), P=0.9279]. The median delta maxDSA of BM-MSCs alone subgroup was -4899 (IQR -8320 to 285.5) while in the BM-MSCs combined with other treatments subgroup was -3824 (IQR -10961 to 1576) (p=0.7618).

### Comparison With the Contemporaneous Control Cohort

There were no significant differences in the baseline characteristics and Banff scores between the total BM-MSCs treatment population and the control cohort (**Tables 1**, **2**). The median delta eGFR of the control cohort was -7.0 ml/min per 1.73m^2^ (IQR -17.1 to 3.1) one year after diagnosis, and -12.7 ml/min/1.73m^2^ (IQR -22.2 to -3.5) after two years (**Figure 1G**). The median eGFR of the control cohort was 25.1 ml/min per 1.73m^2^ (IQR 12.2 to 42.8) one year after diagnosis and 17.2 ml/min per 1.73m^2^ (IQR 0 to 33.7) after two years (**Figure 1H**). In addition, it was observed that eGFR significantly declined more rapidly in the control cohort than that in the BM-MSCs treated group (p=0.4382, year one; p=0.0342, year two) (**Figure 1I**). The graft survival rate after two years of cABMR diagnosis of the control cohort was 66.7%, while in the BM-MSCs treated group, after two years of BM-MSCs treatment, was 87.0% (P=0.1012) (**Figure 3**). Multivariate linear regression was performed to adjust for the confounding effect of baseline eGFR on delta eGFR at two years and the eGFR of the BM-MSCs treated group declined slower (hazard ratio, 8.691; 95% confidence interval, 0.496-16.885; p=0.038) (**Table 4**).

**Table 4:** Effect of BM-MSCs treatment on delta eGFR at two year.

	Univariate analysis		Multivariate analysis	
	HR (95%CI)	P value	HR (95%CI)	P value
Treatment (BM-MSCs *vs* control)	7.284 (-0.875-15.444)	0.079	8.691 (0.496-16.885)	0.038
Pre-eGFR (per 1 ml/min/1.73m^2^)	-0.139 (-0.366-0.088)	0.224	-0.188 (-0.412-0.036)	0.099

BM-MSCs, bone marrow derived mesenchymal stem cells; Pre-eGFR, estimated glomerular filtration rate pre-MSCs treatment or at diagnosis; HR, hazard ratio. 95%CI, 95% confidence interval.

### Changes in Peripheral Blood Lymphocytes Subsets

**Figure 4:**
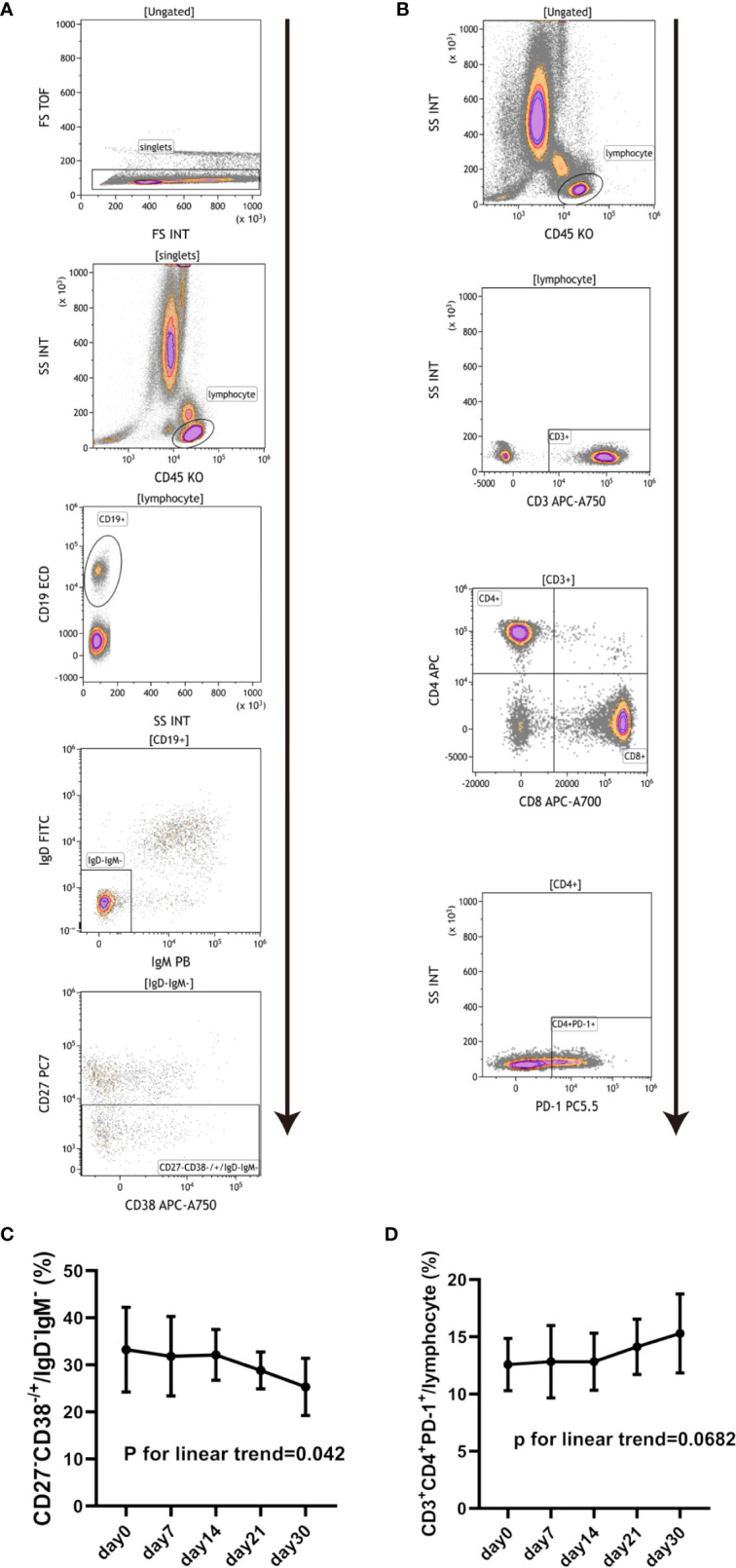
Changes in peripheral blood lymphocyte subsets in cABMR patients after BM-MSCs treatment. Representative flow cytometry plots of CD27^-^CD38^-/+^/IgD^-^IgM^-^ **(A)** and CD3^+^CD4^+^PD-1^+^/lymphocyte **(B)**. Percentage of CD27^-^CD38^-/+^/IgD^-^IgM^-^ pre and after BM-MSCs treatment in one month **(C)**. Percentage of CD3^+^CD4^+^PD-1^+^/lymphocyte pre and after BM-MSCs treatment in one month **(D)**. Points represent mean. Bars represent standard deviation. Data were analyzed by linear trend test.

The changes in peripheral lymphocytes subsets including T cells, B cells, monocytes and NK cells were analyzed in the pre-treatment stage and at days 7, 14, 21, and 30 post-treatment. The blood samples collected belonged to seven patients from Regimen 2 group. **Figures 4A, C** shows that the proportion of CD27^-^CD38^-/+^/IgD^-^IgM^-^ (double negative B cell) decreased after BM-MSCs treatment. In addition, CD3^+^CD4^+^PD-1^+^/lymphocyte (**Figures 4B, D**) also trended to increase in time (although this was not significant). Significant variations were not detected in other representative subsets of T cell (**Supplemental Figure 3-1**), B cells (**Supplemental Figure 3-2**), NK cells (**Supplemental Figure 3-3**), monocytes (**Supplemental Figure 3-3**), dendritic cells (**Supplemental Figure 3-4**), and regulatory T cells (**Supplemental Figure 3-5**) were not found significantly changed.

### Variation in Peripheral Blood Cytokines and Chemokines

**Figure 5:**
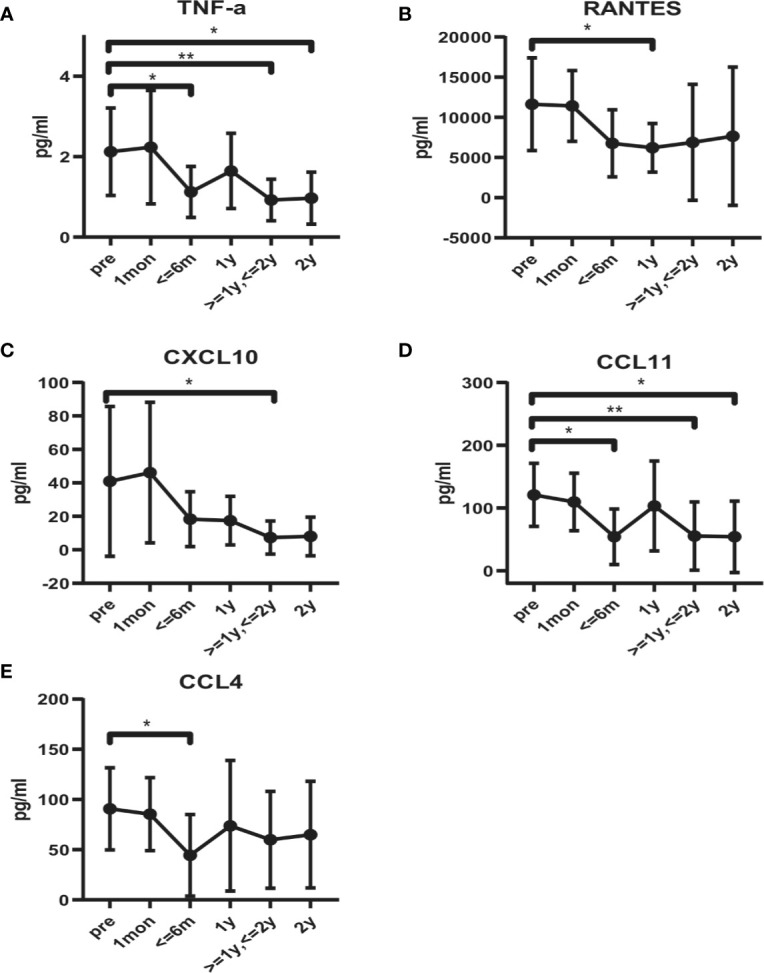
Alteration in serum cytokines and chemokines profile in BM-MSCs treated cABMR patients. Serum levels of TNF-α **(A)**, RANTES **(B)**, CXCL10 **(C)**, CCL11 **(D)**, and CCL4 **(E)**. Points represent mean. Bars represent standard deviation. Data were analyzed by unpaired t-test. *p<0.05, **p<0.01.

Serum concentrations of cytokines and chemokines were examined at different times after BM-MSCs treatment. we found that the concentration of the proinflammatory cytokine TNF-α significantly decreased after four doses of BM-MSCs infusion (**Figure 5A**). The mean level of TNF-α pre-treatment was 2.12 ± 1.09 pg/ml and declined to 1.12 ± 0.63 pg/ml six months after treatment. After two years, TNF-α concentration further decreased to 0.92 ± 0.56 pg/ml. Moreover, RANTES, CXCL10, CCL11 and CCL4 concentrations declined after BM-MSCs treatment (**Figures 5B–E**). The cytokines and chemokines that did not show significant variations are listed in **Supplemental Figures 4-1** and **4-2**.

## Discussion

To the best of our knowledge, this is the first clinical trial demonstrating the efficacy and safety of allogeneic BM-MSCs administration for treatment of cABMR in kidney transplantation. A previous multi-centered study has demonstrated that the eGFR of patients with biopsy-proven late active ABMR significantly declines by a delta value of -9.084 ml/min per 1.73m^2^ one year after cABMR diagnosis (21). Our study found the eGFR of cABMR patients treated with BM-MSCs declined by a median of 2.2 ml/min per 1.73m^2^ and 4.3 ml/min per 1.73m^2^ one and two years after BM-MSCs treatment, respectively. However,the eGFR of the contemporaneous control cohort declined by a median of 12.7 ml/min per 1.73m^2^ two years after cABMR diagnosis. The two-year allograft survival of patients treated with BM-MSCs was higher than that of the contemporaneous control cohort (87.0% *vs*. 66.7%), although the difference did not reach statistical significance. Allogeneic MSCs was previously deemed to increase the risk of sensitization and antibody-mediated allograft rejection (22, 23). Nonetheless, in our study, we found that four doses of intravenous BM-MSCs did neither increase cABMR episodes nor deteriorate renal allograft function when compared to the control cohort and data from a published patient cohort with cABMR (21). Instead, we observed BM-MSCs treatment decelerated the loss of renal allograft function.

It has been previously proven that MSCs could reduce pathogenic antibodies in patients with SLE (24, 25). In agreement, in our study, BM-MSCs treatment significantly reduced DSA level after one and two years of its initial administration. Considering the eGFR was maintained, the results indicate that BM-MSCs presents therapeutic potential for cABMR treatment. In addition, three grafts lost function within two years of follow-up. We retrospectively analyzed the baseline data and found that their eGFR were respectively 25.6, 20.9 and 25.4 ml/min per 1.73m^2^. These results indicate that MSCs may not be suitable for cABMR patients with severely deteriorated renal allograft function. It is of note that many enrolled patients in this study received other treatments for cABMR besides MSCs infusion. To further address the efficacy of MSCs on cABMR, we divided the enrolled patients into two subgroups, i.e. MSCs alone, and MSCs combined with other treatments. These two subgroups were comparable in baseline eGFR and maxDSA, and outcome result showed delta eGFR (-4.3 ml/min/1.73m2 *vs* -3.8 ml/min/1.73m2; p=0.9279) and delta maxDSA (-3824 *vs* -4899; p = 0.7618) were not significant different between these two subgroups. This result indicates the combined treatment administered before or after MSCs may not affect the evaluation of MSCs efficacy on cABMR in this study.

A clinical study has reported the safety of third-party BM-MSCs administration among kidney transplantation recipients (16). The only reported adverse event was the non-ST elevation of myocardial infarction hours after MSCs infusion in one patient with ischemic heart disease and postoperative anemia. Another publication reported the safety of multiple injection of third-party BM-MSCs for treatment of two kidney transplant patients with refractory cABMR, and only diarrhea and hypertension were observed, while the renal allografts deteriorated six months after MSCs treatment (26). Herein, we observed four cases of elevated hepatic enzymes, three of which resulted from replication of pre-existing HBV or HCV. Moreover, BKV and CMV infections were also observed in our study. In accordance, BKV and CMV infections were also reported by Erpicum et al., but there was no difference compared with the concurrent controls (16). Furthermore, no fever, anaphylaxis, phlebitis or venous thrombosis, cardiovascular complications or malignancy were identified in our study. The results indicate the safety of multiple doses of third-party BM-MSCs infusion in kidney recipients with cABMR.

Previous studies have demonstrated MSCs can modulate the humoral immunity by inhibiting the maturation and proliferation of B cells, modulating their activation and inducing regulatory B cells (Breg) (27–30). An *in vivo* study has showed that administration of MSCs combined with rapamycin generates suppression of antibody production and B cell proliferation (31). In this sense, clinical studies have confirmed that MSCs can induce Breg in human (32). Furthermore, MSCs have been used to treat systemic lupus erythematosus (SLE), rheumatoid arthritis and other autoimmune diseases. In this study, we conducted short-term immunosurveillance after BM-MSCs treatment. Immune monitoring was performed according to the methods validated in “The One Study” as described by Streitz et al., with minor modification (33). We found CD27^-^CD38^-/+^/IgD^-^IgM^-^ (Double Negative B cells, DN B cells) population trended to decrease in a dose-dependent manner after BM-MSCs treatment. DN B cells have been recently describe as a novel memory B cells population lacking CD27 expression, a generally used memory B cells marker which expression correlates to somatic mutations in the immunoglobulin genes (34, 35). These B cells do not express IgD indicating that they have undergone class switching, although they do not gain CD27 expression. DN B cells are suggested to be the precursors of antibody secreting cells in SLE patients and to develop outside the germinal center (36, 37). While DN B cells can be detected in healthy individuals, they expand in patients with SLE (38), rheumatoid arthritis (39), non-small cell lung cancer (40), and COVID-19 (41). Furthermore, it has been reported that DN B cells increased in pediatric acute renal allograft rejection recipients who received steroid pulsing, compared to patients with stable graft function (42). The expansion of this cell population has also been observed in pediatric kidney transplant recipients who developed anti-HLA antibodies (43). It has been reported to correlated with poor response to B cell depletion therapy and active disease in SLE (34, 36, 44). In addition, DN B cells can contribute excess of 40% of all B cells in active SLE and may become the largest circulating population of isotype switched IgD^–^ cells (45). Among these expanded DN B cells, most were DN2 B cells (CXCR5^-^ CD11c^+^), which representing pre-plasma cells (36). Thus, we speculated that BM-MSCs plays a role in the treatment of cABMR by decreasing DN B cells, probably DN2 B cells. We also found that the proportion of CD3^+^CD4^+^PD-1^+^/lymphocyte tended to increase after BM-MSCs infusion. Programmed cell death protein 1 (PD-1) is an important costimulatory molecule that involved in the downregulation of activated T cells (46, 47). The T cells that upregulate PD-1 expression show an exhausted state and functional unresponsiveness, preventing massive immunoactivation (48). Therefore, this might indicate that BM-MSCs could modulate the immune status in cABMR patients by inducing PD-1 positive CD4+ T cells.

MSCs can orchestrate the inflammatory state of microenvironments by modulating the secretion of multiple components including cytokines, chemokines, anti-inflammatory mediators and exosomes, which exert immunoregulatory effects on various immune cells (49–51). Our study showed that the proinflammatory cytokine TNF-α and the chemokines CXCL10, CCL4, CCL11 and RANTES decreased after BM-MSCs treatment. Previous studies have found that TNF, CXCL10, CCL4, and RANTES are among the prime genes of ABMR, which are the potential diagnostic and prognostic molecular endpoints and biomarkers for clinical trials recommended by the Banff 2017 kidney meeting report (52). This indicated that BM-MSCs administration may alleviate inflammatory responses in cABMR patients.

The study is subjected to some limitations. This is a single-arm and single-center study with small sample size. The pathological change of renal allografts was not examined since protocol biopsy was unable to be performed. This prospective phase I/II study demonstrated the promising potential of MSCs for treatment of cABMR in kidney transplantation, and a large well-designed study is advocated to further determine its efficacy.

In conclusion, this study demonstrated that immunosuppressive drugs combined with intravenous administration of BM-MSCs decrease DSA and facilitates the maintenance of renal allograft function for kidney transplant recipients with chronic active ABMR without significant adverse events. The underlying mechanisms might involve immunomodulatory effect of MSCs on peripheral CD27^-^IgD^-^ B cells and CD4^+^PD-1^+^ T cells.

## Data Availability Statement

The raw data supporting the conclusions of this article will be made available by the authors, without undue reservation.

## Ethics Statement

This study was reviewed and approved by the Ethics Committee and the Research Board, the First Affiliated Hospital of Sun Yat-sen University and was in accordance with the principles of the World Medical Association Declaration of Helsinki. The patients receiving BM-MSCs treatment provided their written consent to participate this study. Consent of patients from the contemporaneous control cohort was waived.

## Author Contributions 

As for individual contribution, YW, LL, and HZ, study design, data analysis and interpretation and manuscript preparation. XC, GL, XZ, and APX, MSCs preparation and quality control. LL, QF, JL, RD, CXW, and CLW, patient enrollment and informed consent. QS, XL, and QY, laboratory examination and data analysis. SY, pathology examination. YP and YG, academic consult. JW and XS, clinical data collection. HH and BX, sample collection. APX and CXW, funding support, study supervision and critical review of manuscript. CXW, APX and LL are responsible for the decision to submit for publication. All authors contributed to the article and approved the submitted version.

## Funding

This study was supported by Science and Technology Planning Project of Guangdong Province, China (2015B020226002, 2014B020212006, 2017A020215012), National Natural Science Foundation of China (81870511, 81670680, 81700655, 81970109, 81300623), Key Scientific and Technological Program of Guangzhou City (201803040011), Guangdong Basic and Applied Basic Research Foundation (2020A1515010884), Guangdong Natural Science Foundation (2018A030313016), Pearl River S&T Nova Program of Guangzhou (201906010095), Guangdong Provincial Key Laboratory on Organ Donation and Transplant Immunology (2013A061401007, 2017B030314018), and Guangdong Provincial International Cooperation Base of Science and Technology (Organ Transplantation, 2015B050501002).

## Conflict of Interest

The authors declare this study was conducted in the absence of any commercial or financial relationships that could be construed as a potential conflict of interest.
