# Supplementary material for: Efficacy and Safety of Bone Marrow-Derived Mesenchymal Stem Cells for Chronic Antibody-Mediated Rejection After Kidney Transplantation- A Single-Arm, Two-Dosing-Regimen, Phase I/II Study
Source: Front Immunol. 2021 Jun 25;12:662441. doi: 10.3389/fimmu.2021.662441 (PMC8267917; doi:10.3389/fimmu.2021.662441)
Supplement: Supplementary file 1 [file DataSheet_1.docx]

Supplementary Material

# Supplementary Data

**Supplemental table 1**. Other medications for renal chronic antibody-mediated rejection of patients treated with BM-MSCs.

| Patient ID | Before BM-MSCs treatment (days) | After BM-MSCs treatment (days) |
| --- | --- | --- |
| 1 | MP+PP+IVIG+ rituximab (77) | - |
| 2 | - | - |
| 3 | MP+PP+IVIG+ rituximab (94) | IVIG (539) |
| 4 | - | - |
| 5 | - | - |
| 6 | IVIG+ rituximab (9) | IVIG (435) |
| 7 | PP+IVIG+ rituximab (21) | - |
| 8 | - | - |
| 9 | - | IVIG (36) |
| 10 | - | - |
| 11 | MP+IVIG+ rituximab + bortezomib (361) | - |
| 12 | - | - |
| 13 | - | - |
| 14 | IVIG (825) | - |
| 15 | - | - |
| 16 | - | - |
| 17 | MP+PP+IVIG (42) | - |
| 18 | - | IVIG (91) |
| 19 | - | - |
| 20 | - | MP+IVIG+ rituximab (28) |
| 21 | - | - |
| 22 | MP+PP (58) | - |
| 23 | - | - |

BM-MSCs, bone marrow derived mesenchymal stem cells; MP, methylprednisolone; IVIG, intravenous immunoglobulin; PP, plasmapheresis.

**Supplemental table 2.** Commerial flow cytometry panels used for peripheral blood lymphocyte immunophenotyping.

| Panel | Marker (fluorescein) |
| --- | --- |
| DuraClone® IM Phenotyping Basic panel | CD16-FITC, CD56-PE, CD19-ECD, CD14-PC7, CD4-APC, CD8-A700, CD3-APC-A750 and CD45-Krome Orange |
| DuraClone® IM B cell panel | IgD-FITC, CD21-PE, CD19-ECD, CD27-PC7, CD24-APC, CD38-APC-A750, IgM-Pacific Blue and CD45-Krome Orange |
| DuraClone® IM T Cell Subsets panel | CD45RA-FITC, CCR7-PE, CD28-ECD, PD-1-PC5.5, CD27-PC7, CD4-APC, CD8-A700, CD3-APC-A750, CD57-Pacific Blue and CD45-Krome Orange |
| DuraClone® IM Dendritic Cell panel | CD16-FITC, Lineage-PE, CD1c-PC5.5, CD11c-PC7, Clec 9A-APC, CD123-APCA700, HLA-DR-Pacific Blue and CD45-Krome Orange |
| DuraClone® IM Treg panel | CD45RA-FITC, CD25-PE, CD39-PC5.5, CD4-PC7, Foxp3-A647, CD3-APC-A750, Helios-Pacific Blue and CD45-Krome Orange |
| DuraClone® IM TCRs panel | TCR γδ-FITC, TCR αβ-PE, HLA-DR-ECD, TCR Vδ1-PC7, CD4-APC, CD8-A700, CD3-APC-A750, TCR Vδ2-Pacific Blue and CD45-Krome Orange |

**Supplemental Figure Legends**

**Supplemental Figure 1. Biological characteristics of BM-MSCs.** BM-MSCs cell morphology **(A)**, representative growth curve **(B),** and flow cytometric analysis of surface markers **(C)**.

**Supplemental Figure 2. Gating strategy for six flow cytometry panels used for blood lymphocyte immunophenotyping.** Representative flow cytometry analysis plots for DuraClone® IM B cell panel **(A)**, DuraClone® IM Phenotyping Basic panel **(B)**, DuraClone® IM T Cell Subsets panel **(C)**, DuraClone® IM Treg panel **(D)**, DuraClone® IM TCRs panel **(E)**, and DuraClone® IM Dendritic Cell panel **(F)**.

**Supplemental Figure 3-1 to 3-5. Changes in the relative proportion of representative subsets of T cells, B cells, NK cells, monocytes, dendritic cells and regulatory T cells (Treg) analyzed by multicolor flow cytometry.** T cells subsets **(3-1)**, B cells subsets **(3-2)**, NK cells subsets **(3-3)**, monocyte and subsets **(3-3)**, dendritic cells subsets **(3-4)**, Treg subsets **(3-5)**. Points represent mean. Bars represent standard deviation. Data were analyzed by unpaired t-test.

**Supplemental Figure 4-1, 4-2. Cytokines and chemokines concentrations measured in BM-MSCs treated cABMR patients.** Cytokine and chemokine expression levels were determined using a multiplex cytokine assay. Points represent mean. Bars represent standard deviation. Data were analyzed by unpaired t-test.
