# Supplementary material for: Efficacy and Safety of Bone Marrow-Derived Mesenchymal Stem Cells for Chronic Antibody-Mediated Rejection After Kidney Transplantation- A Single-Arm, Two-Dosing-Regimen, Phase I/II Study
Source: Front Immunol. 2021 Jun 25;12:662441. doi: 10.3389/fimmu.2021.662441 (PMC8267917; doi:10.3389/fimmu.2021.662441)
Supplement: Supplementary file 2 [file DataSheet_2.pdf]

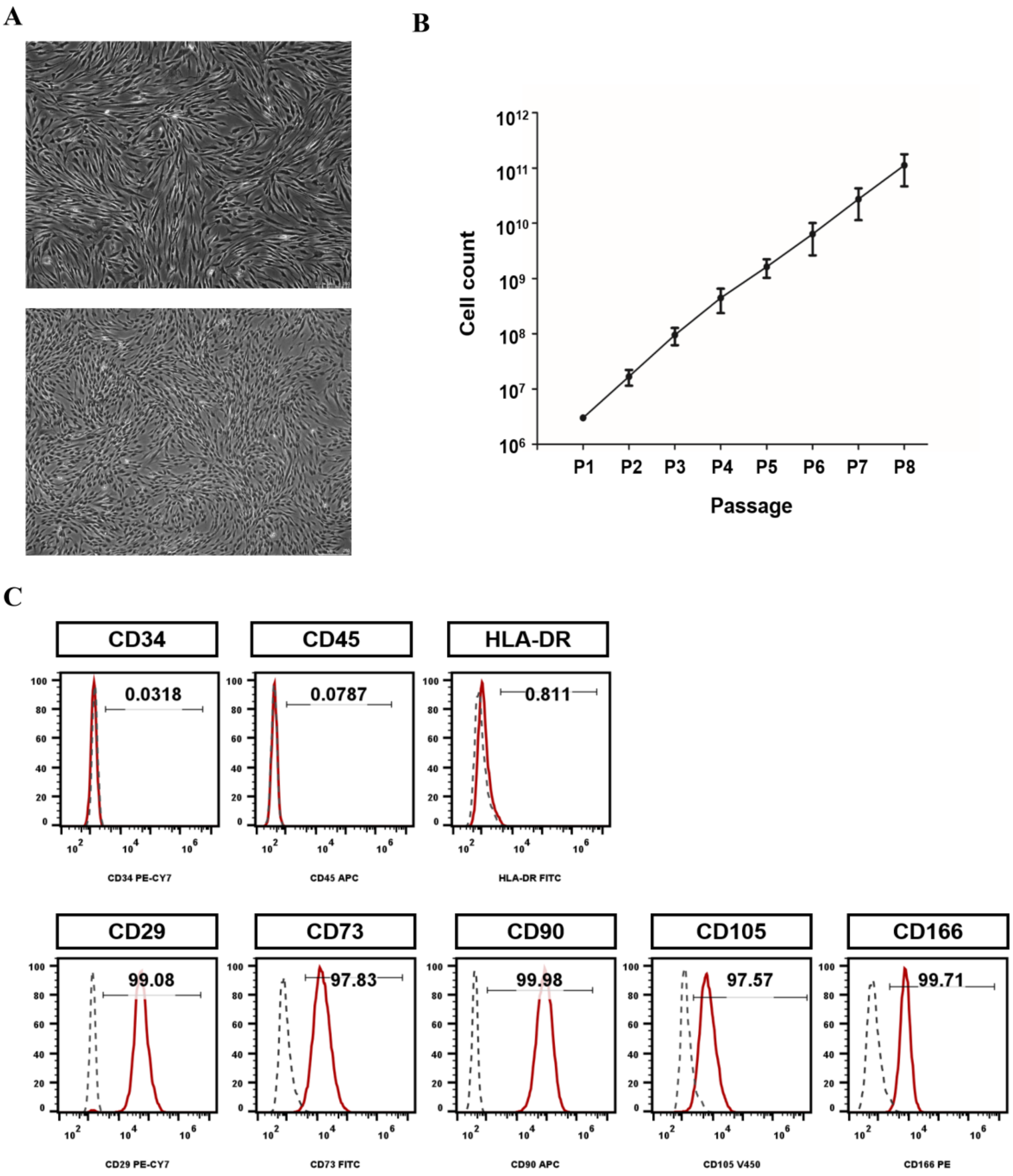

Supplemental Figure 1

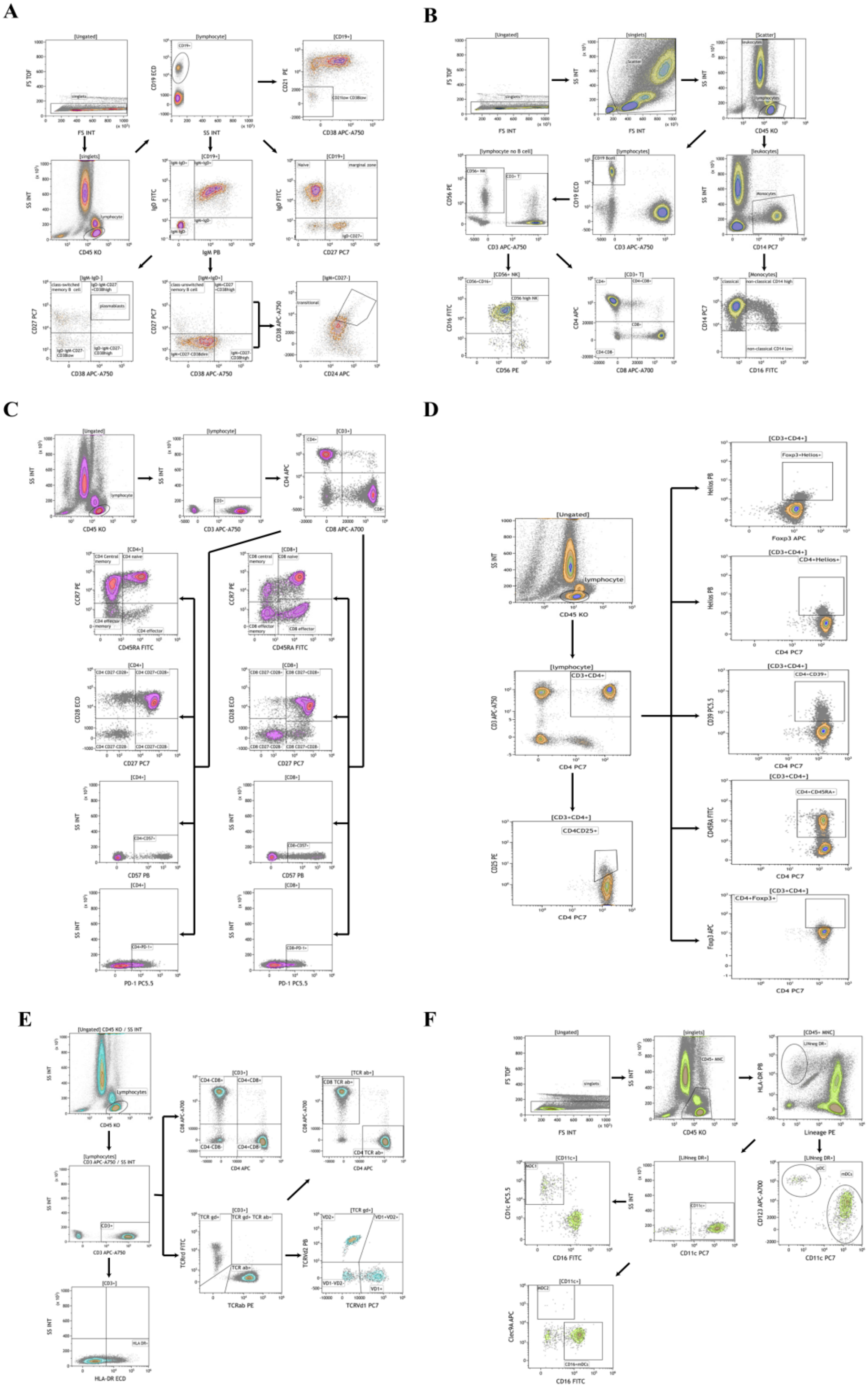

Supplemental Figure 2

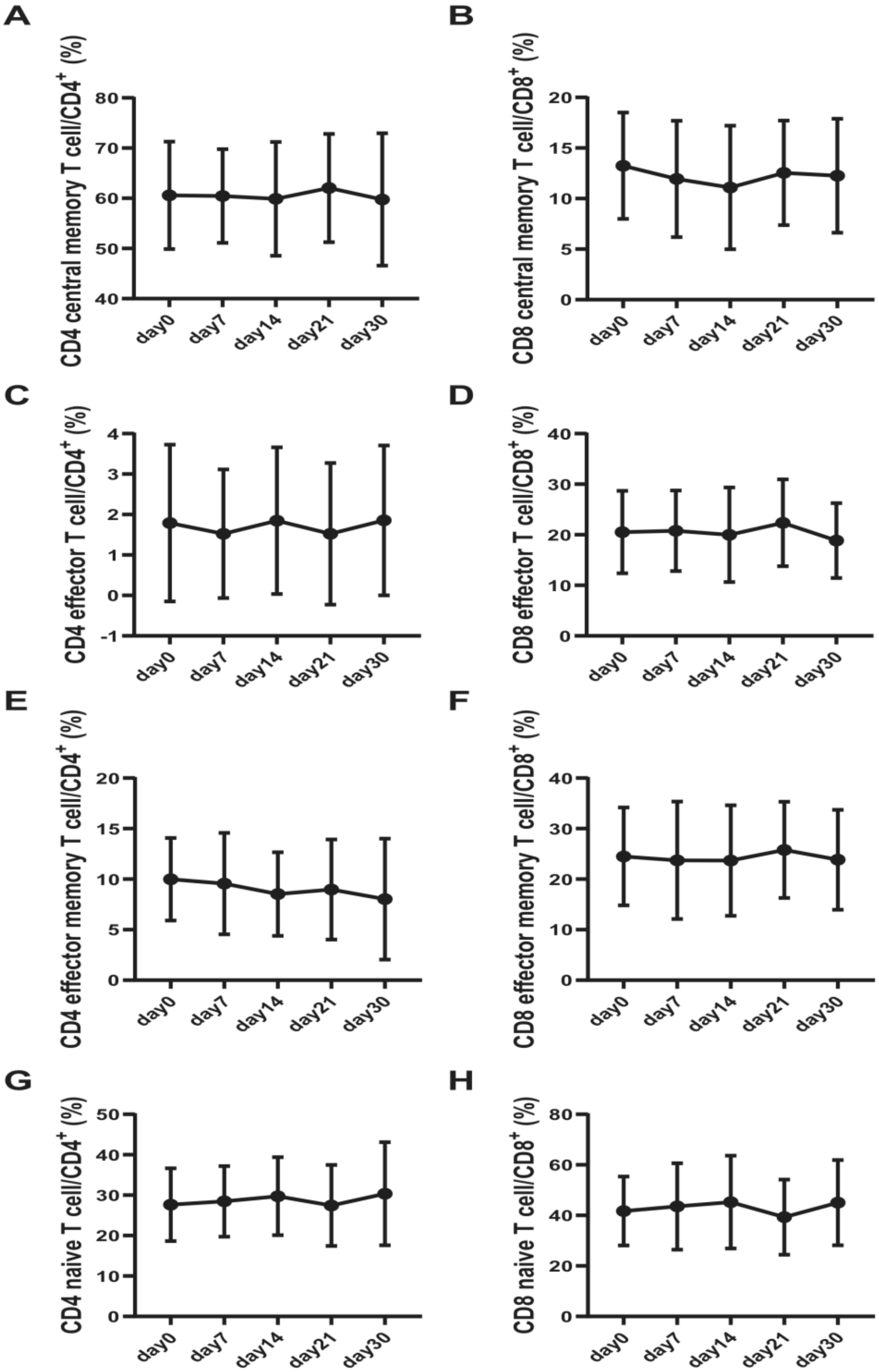

Supplemental Figure 3-1

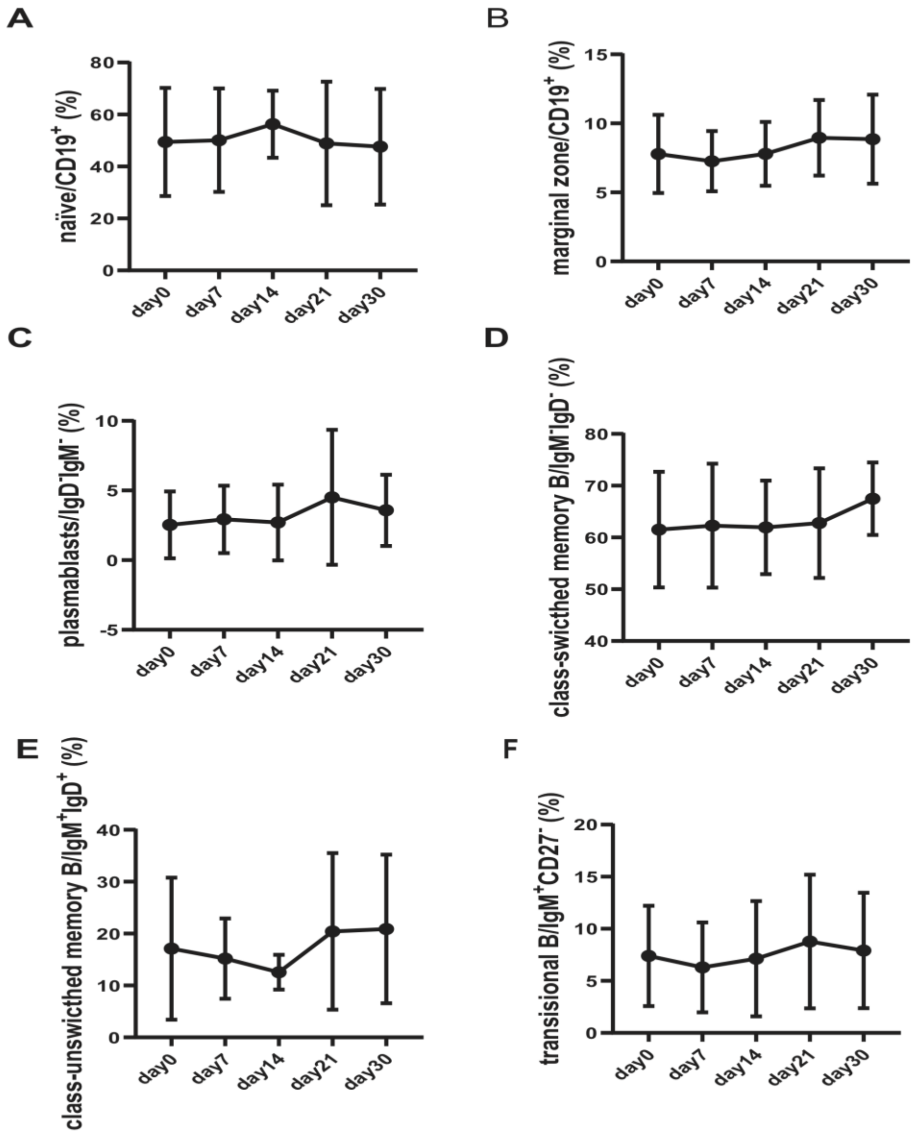

Supplemental Figure 3-2

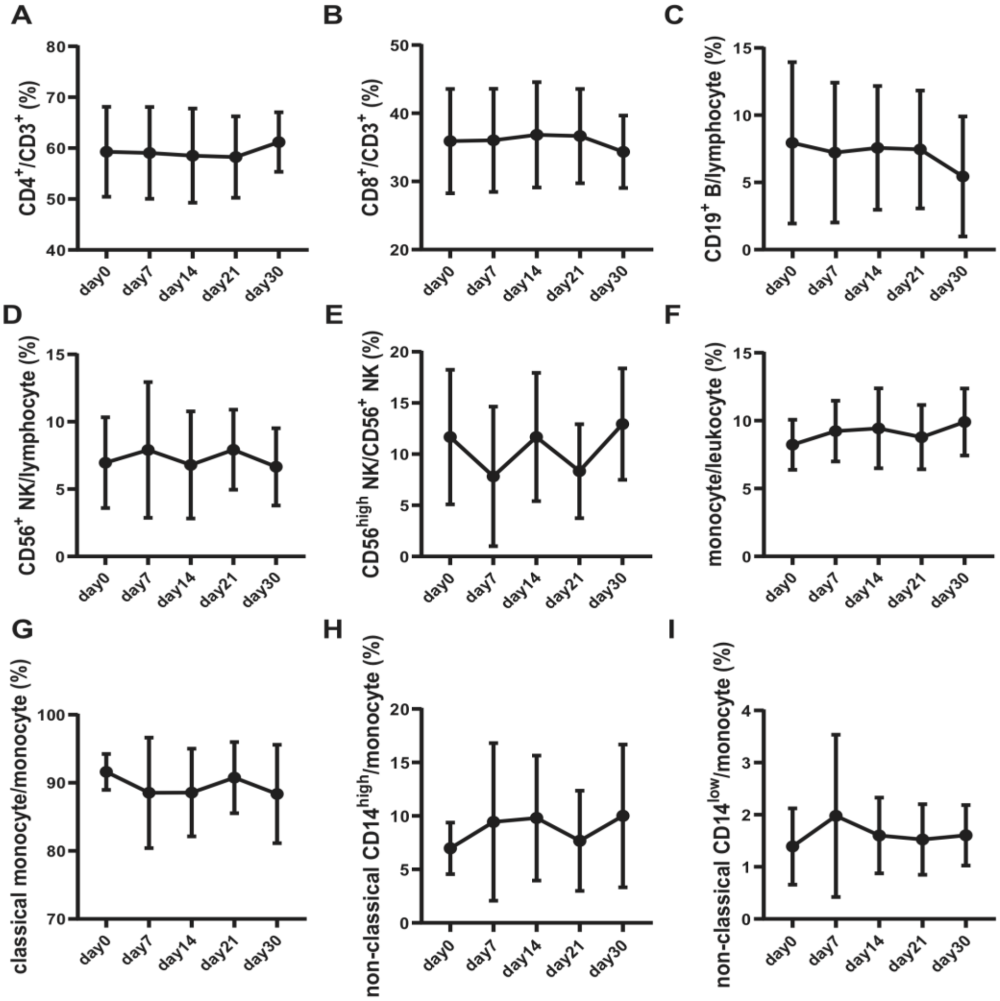

Supplemental Figure 3-3

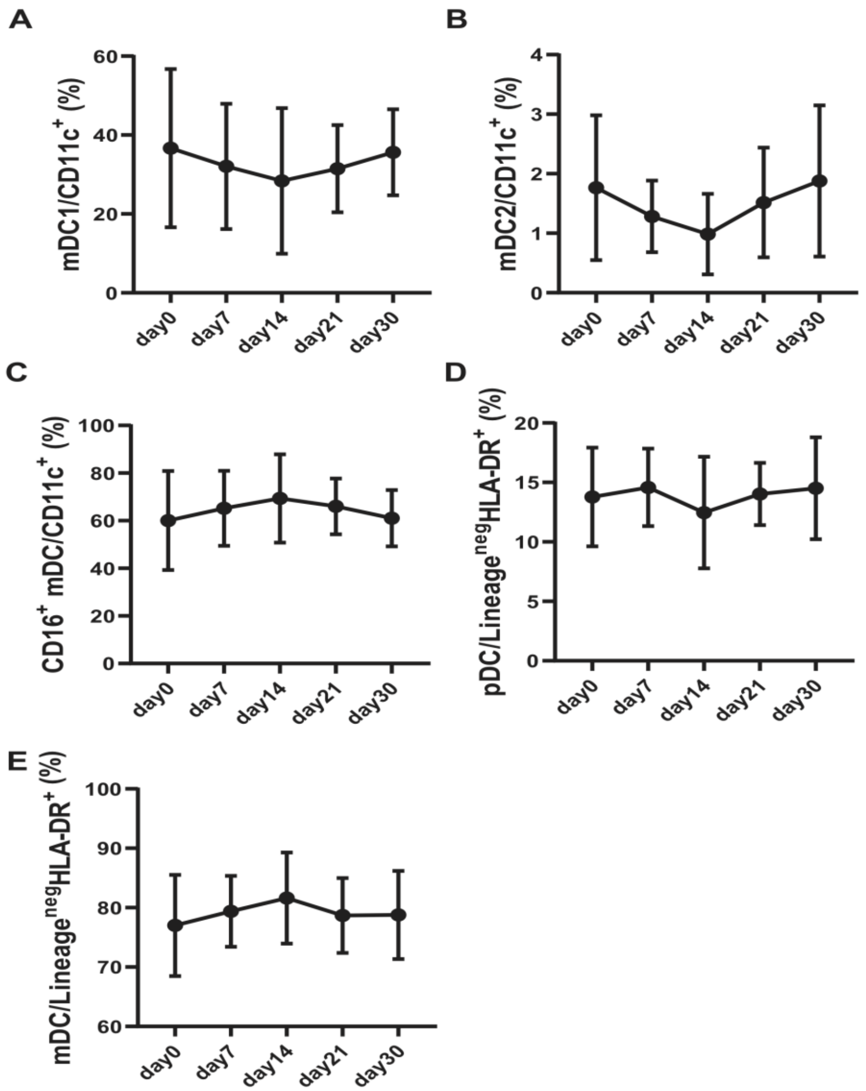

Supplemental Figure 3-4

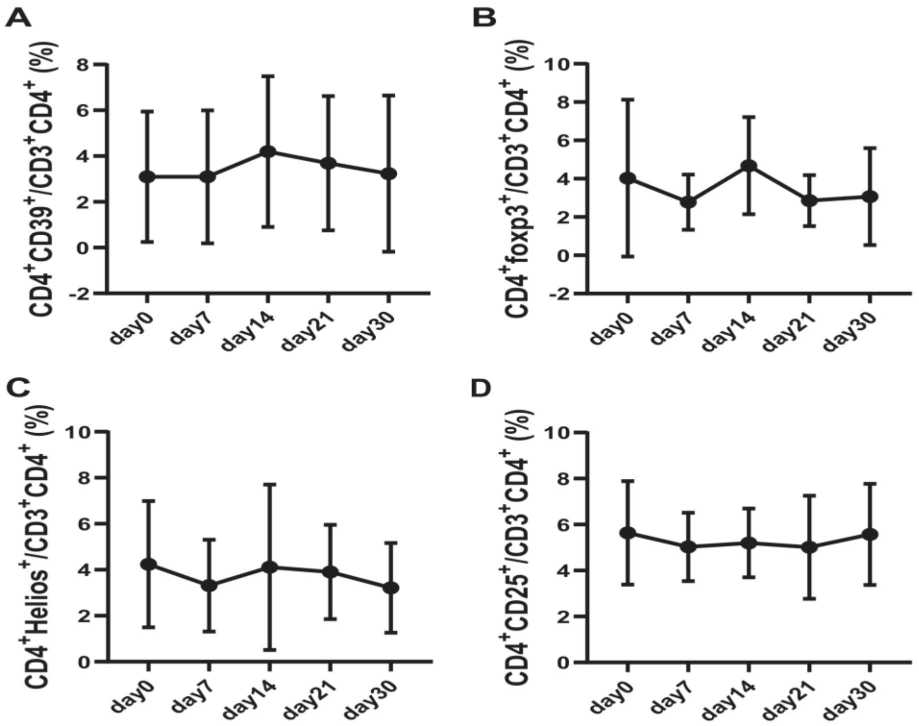

Supplemental Figure 3-5

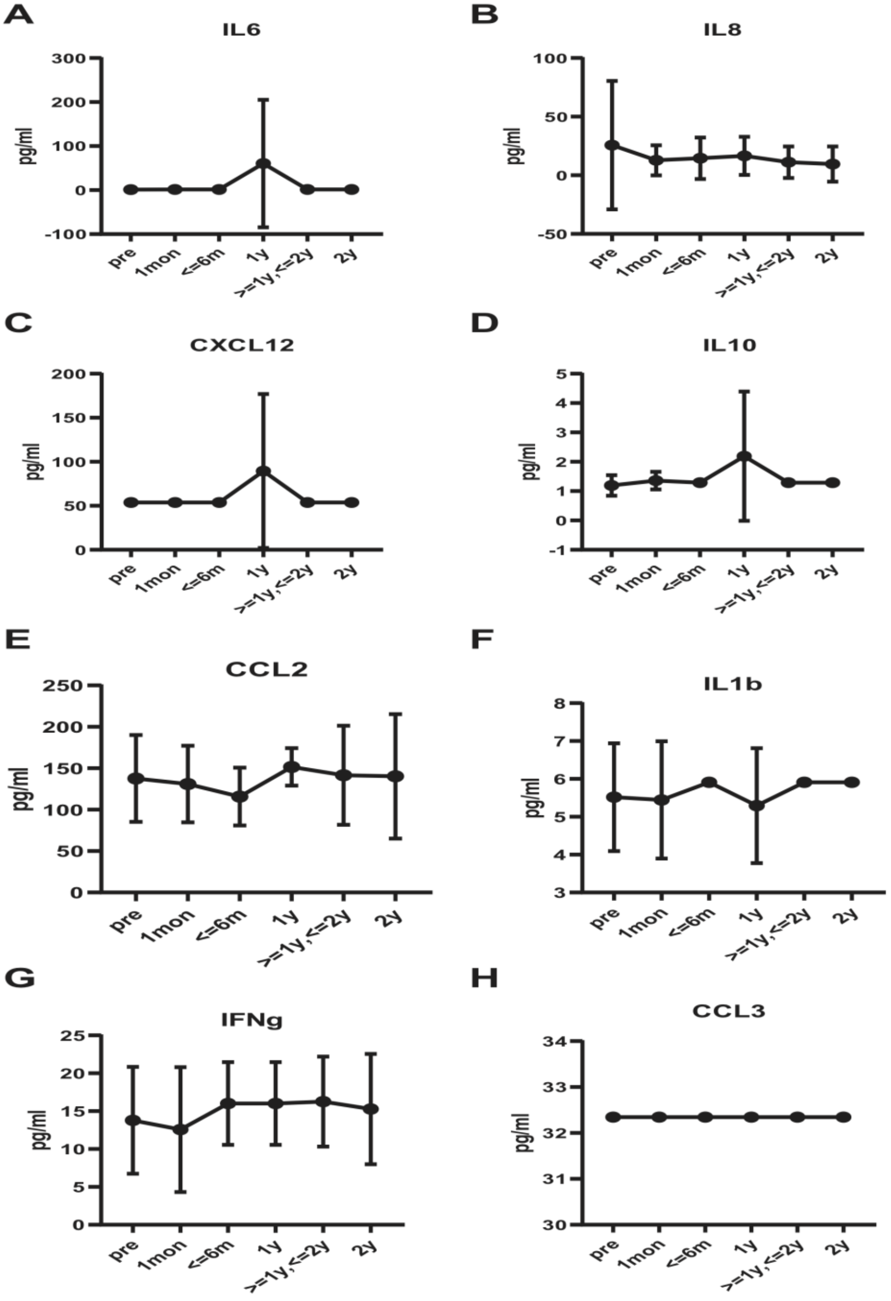

Supplemental Figure 4-1

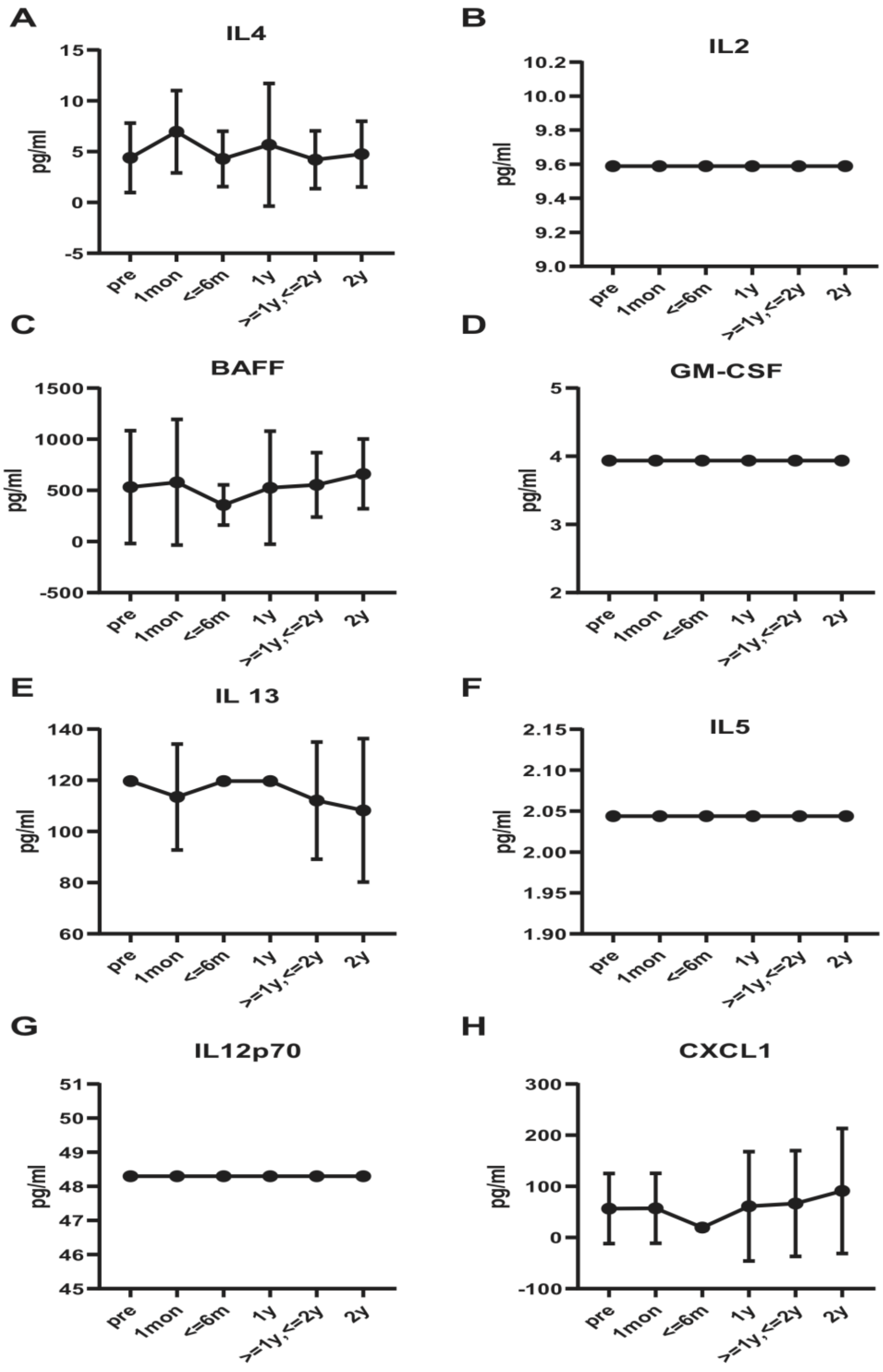

Supplemental Figure 4-2
